# Off-Design Operation and Cavitation Detection in Centrifugal Pumps Using Vibration and Motor Stator Current Analyses

**DOI:** 10.3390/s24113410

**Published:** 2024-05-25

**Authors:** Yuejiang Han, Jiamin Zou, Alexandre Presas, Yin Luo, Jianping Yuan

**Affiliations:** 1 Research Center of Fluid Machinery Engineering and Technology, Jiangsu University, Zhenjiang 212013, China; kmusthyj@163.com (Y.H.); 2112111019@stmail.ujs.edu.cn (J.Z.); luoyin@ujs.edu.cn (Y.L.); yh@ujs.edu.cn (J.Y.); 2 Centre for Industrial Diagnostics and Fluid Dynamics, Polytechnic University of Catalonia, 08034 Barcelona, Spain

**Keywords:** centrifugal pump, off-design operation, cavitation, three-axis accelerometer, Hall-effect current sensors, vibration, motor stator current, machine learning

## Abstract

Centrifugal pumps are essential in many industrial processes. An accurate operation diagnosis of centrifugal pumps is crucial to ensure their reliable operation and extend their useful life. In real industry applications, many centrifugal pumps lack flowmeters and accurate pressure sensors, and therefore, it is not possible to determine whether the pump is operating near its best efficiency point (BEP). This paper investigates the detection of off-design operation and cavitation for centrifugal pumps with accelerometers and current sensors. To this end, a centrifugal pump was tested under off-design conditions and various levels of cavitation. A three-axis accelerometer and three Hall-effect current sensors were used to collect vibration and stator current signals simultaneously under each state. Both kinds of signals were evaluated for their effectiveness in operation diagnosis. Signal processing methods, including wavelet threshold function, variational mode decomposition (VMD), Park vector modulus transformation, and a marginal spectrum were introduced for feature extraction. Seven families of machine learning-based classification algorithms were evaluated for their performance when used for off-design and cavitation identification. The obtained results, using both types of signals, prove the effectiveness of both approaches and the advantages of combining them in achieving the most reliable operation diagnosis results for centrifugal pumps.

## 1. Introduction

Centrifugal pumps are vital segments in fluid transmission and energy storage. A centrifugal pump is designed to achieve the best efficiency point (BEP) using a specific combination of capacity, head, and speed [1]. Detecting and evaluating abnormal operation conditions before they lead to catastrophic consequences can significantly reduce the risks associated with the productive chain. Therefore, the operation diagnosis of centrifugal pumps has become one of the most important tasks to ensure their stable operation.

In recent research, centrifugal pumps have been diagnosed mainly by vibration-based techniques. Signal processing and machine learning-based algorithms have provided qualified results. Mousmoulis, G. et al. [2] employed the spectral kurtosis tool (SKT) to extract the vibration features induced by impulsive shock waves. They correlated the periodic impulsive features extracted by SKT with the blade passing frequency, which is useful for detecting faults in centrifugal pumps. In [3], Sun, H. et al. presented a strategy for detecting seal damage and cavitation in centrifugal pumps. A calculation of cyclic autocorrelation functions (CAFs) for the vibration signals was conducted to extract the characteristic frequency components corresponding to centrifugal pump faults. In [4], Zhang, M. et al. employed variational mode decomposition (VMD) to extract the principal mode of the vibration signals in a multistage centrifugal pump to detect bearing faults. In [5], the Markov parameters calculated from the vibration signals were used as features in classification algorithms based on convex optimization to detect incipient cavitation in a centrifugal pump. The algorithm was validated to achieve high accuracy within a relatively limited budget. In [6], an integrated vibration-based centrifugal pump fault-diagnosis framework based on the combination of wavelet transform and principal component analysis (PCA) was developed, which performs well in the detection of impeller faults, bearing faults, and blockages for centrifugal pumps. In [7], Kumar, A. et al. combined noise subtraction and the marginal enhanced square envelope spectrum for detecting bearing defects in both centrifugal pumps and axial pumps. In [8], Wu, K. et al. used the Enkurgram to extract characteristic frequencies for fluid machinery. It has been proved that the proposed method has better demodulation capability in dealing with vibration signals with a low signal-to-noise ratio from centrifugal pumps. 

In recent decades, there has been an increasing need for easy-to-install systems to monitor centrifugal pumps, which limits the application of vibration sensors in practical engineering cases. Meanwhile, vibration sensors are susceptible to environmental disturbances, which could reduce the measurement accuracy and lead to misdiagnosis [9]. In the 1970s, the concept of motor current signature analysis (MCSA) was first proposed to monitor inaccessible motors placed in nuclear power plants [10]. In this technique, the stator current characteristics directly linked to the instantaneous change of rotating flux caused by mechanical or electrical faults can be used for fault detection. Applications of MCSA have been reported to be cheaper without the need to access the motor. Current transformers can be implemented inexpensively and non-intrusively in almost all applications [11]. Multiple devices can be monitored simultaneously in a remote power distribution room based on MCSA. There has been extensive research on MCSA, mostly focusing on the fault detection of motors [12,13,14]. As for the applications of MCSA in centrifugal pumps, in [15], Harihara, P.P. et al. automated a method for the detection of the onset of pump cavitation based on the line voltage and phase current. They built a predicted model of the stator current of the pump and used the residuals between the measured and predicted current for detection so that the interference of the electric power supply could be avoided. In [16], Hernandez-Solis, A. et al. used current and power signature analyses as diagnostic tools for submersible centrifugal pumps. It is mentioned that the effects of cavitation are well appreciated using both techniques by tracking the characteristic harmonics corresponding to pump performance. In [17], Alabied, S. et al. used intrinsic time scale decomposition (ITD) for feature extraction and later evaluated the health condition of a centrifugal pump with a support vector machine (SVM) based on a directed acyclic graph (DAG) using the stator current signals. In [18], Popaleny, P. et al. outlined an expert diagnostic system for cryogenic pumps based on motor voltage and current signals, which was able to distinguish automatically among different faults with high immunity to noise and power fluctuation. In [19], Hilbert–Huang Transform (HHT) was used to demodulate and denoise the current signals. Time-frequency characteristics extracted from the current signals using HHT were selected to indicate the cavitation severity in a centrifugal pump. In [20], Husna, A. et al. built a discriminative feature pool to detect faults in a centrifugal pump, whose inputs were the cross-correlation of the current signals and motor bearing speed vibration signals. Their work explores the use of different signals together. In [21], Sun, H. et al. utilized complete ensemble empirical mode decomposition with adaptive noise to extract indicators, revealing the energy variation of stator current induced by cavitation, which improves the feasibility of MCSA for cavitation detection in centrifugal pumps. In [22], Sunal, C. et al. used a combination of DQ transformation and transfer learning to detect faults in centrifugal pumps: they fed a DQ image of current signals into a residual network and reached a relatively high classification accuracy. In [23], Zou, J. et al. applied singular-value decomposition and Hilbert–Huang transform to analyze the stator current characteristics of a centrifugal pump, and the maximum amplitude of the Hilbert marginal spectrum was used as an indicator to detect mechanical seal failures.

Fault diagnosis for centrifugal pumps based on vibration and current signals has been well investigated but not to the same extent as off-design operation detection [24]. Centrifugal pumps operating in off-design conditions would cause additional hydraulic torque disturbance forces, and the internal flow status of centrifugal pumps would become unstable, with excessive turbulent disturbance generated. Recirculation below the BEP and flow separation at high flows above the BEP would greatly increase the risk of damage when pumps work continuously [1]. Most of the existing literature focuses on unique cases of detecting mechanical or electrical faults. Fault features are usually extracted by verifying the magnitudes of characteristic frequency components using fast Fourier transform (FFT) or joint time-frequency analysis methods [25]. These features may not be obvious under off-design operation conditions. Therefore, for a real industrial application, it is necessary to develop robust intelligent diagnostic methods to accurately detect either faults or off-design operations for centrifugal pumps.

With the above perspective, this paper uses a three-axis accelerometer and three Hall-effect current sensors to collect signals and demonstrates the combination of vibration and motor stator current analyses to achieve a maximum reliable detection of off-design operation and cavitation in centrifugal pumps. The presented research has the following innovations:

(1) It combines signal processing methods with intelligent classifiers from different families to train a robust operation diagnostic system. The resulting method is validated in a centrifugal pump and proven to be effective.
(2) The operation diagnosis strategy is capable of detecting off-design operation and evaluating the severity of cavitation in centrifugal pumps.

## 2. Theoretical Analysis

The torque oscillations in centrifugal pumps due to turbulent disturbance strongly depend on the flow condition. The design operation condition provides the most stable flow status for centrifugal pumps, and the blade interaction with vortices in radial and axial clearances is the primary source of pump structure vibration and torque oscillations [26]. Part-load conditions (usually when q* = *Q*/*Q_des_* < 0.4~0.75) generate additional turbulent disturbance related to the reciprocal action of recirculation and flow separation. The hydraulic excitation strengthens the pressure pulsation and alternating stresses in various pump components. Under higher flow-rate conditions (q* > 1.1), recirculation is not observed [1]. However, laminar and turbulent boundary layer vortex shedding leads to an increase in flow turbulence. In cavitation conditions, the subsequent implosion of vapor bubbles and cavity fluctuations excite low-frequency pulsation and fluid-borne noise. These hydraulic sources cause vibration and additional torque oscillations in centrifugal pumps. In [27], it is also shown that the hydraulic torque oscillations vary with the flow condition.

Most centrifugal pumps are driven by induction motors. The voltage equation of a three-phase induction motor in the *d-q* coordinate system is given as [28]. In the d-q coordinate system, the *d*-axis is perpendicular to the *q*-axis. The rotational speed of the coordinate system is equal to the synchronous speed of the motor. This transformation from *ABC* to a *d-q* coordinate system simplifies the analysis and control of motor behavior.

(1) $$\left[\begin{array}{c} \begin{array}{c} u_{sd}\\ u_{\mathrm{sq}} \end{array}\\ \begin{array}{c} u_{\mathrm{rd}}\\ u_{\mathrm{rq}} \end{array} \end{array}\right]=\left[\begin{array}{cc} \begin{array}{c} R_{s}\\ \begin{array}{c} 0\\ 0\\ 0 \end{array} \end{array} & \begin{array}{cc} \begin{array}{c} 0\\ R_{s}\\ \begin{array}{c} 0\\ 0 \end{array} \end{array} & \begin{array}{cc} \begin{array}{c} 0\\ 0\\ \begin{array}{c} R_{r}\\ 0 \end{array} \end{array} & \begin{array}{c} 0\\ 0\\ \begin{array}{c} 0\\ R_{r} \end{array} \end{array} \end{array} \end{array} \end{array}\right]\left[\begin{array}{c} i_{\mathrm{sd}}\\ \begin{array}{c} i_{sq}\\ i_{\mathrm{rd}}\\ i_{\mathrm{rq}} \end{array} \end{array}\right]+\frac{d}{dt}\left[\begin{array}{c} \psi_{\mathrm{sd}}\\ \begin{array}{c} \psi_{sq}\\ \psi_{\mathrm{rd}}\\ \psi_{\mathrm{rq}} \end{array} \end{array}\right]+\left[\begin{array}{c} -\omega_{1}\psi_{\mathrm{sq}}\\ \begin{array}{c} {\omega_{1}\psi }_{\mathrm{sd}}\\ {-(\omega_{1}-\omega )\psi }_{\mathrm{rq}}\\ {(\omega_{1}-\omega )\psi }_{\mathrm{rd}} \end{array} \end{array}\right]$$

where *R_s_* is the stator resistance and *R_r_* is the rotor resistance. In the *d-q* coordinate system, *u_sd_* and *u_sq_* are the stator voltages; *u_rd_* and *u_rq_* are the rotor voltages; and *u_rd_* = *u_rq_* = 0 for cage rotors. *i_sd_* and *i_sq_* are the stator currents. *i_rd_* and *i_rq_* are the rotor currents. *ψ_sd_* and *ψ_sq_* are the stator fluxes. *ψ_rd_* and *ψ_rq_* are the rotor fluxes. *ω*_1_ is the synchronous speed. *ω* is the rotational speed.

The flux linkage equation is given as

(2) $$\left\{\begin{array}{c} \begin{array}{c} \psi_{\mathrm{sd}}=L_{s}i_{\mathrm{sd}}+L_{m}i_{\mathrm{rd}}\\ \psi_{sq}=L_{s}i_{sq}+L_{m}i_{\mathrm{rq}} \end{array}\\ \psi_{\mathrm{rd}}=L_{r}i_{\mathrm{rd}}+L_{m}i_{\mathrm{sd}}\\ \psi_{\mathrm{rq}}=L_{r}i_{\mathrm{rq}}+L_{m}i_{sq} \end{array}\right.$$

where *L_s_* is the stator inductance, *L_r_* is the rotor inductance, and *L_m_* is the mutual inductance.

The electromagnetic torque of a three-phase induction motor is given by

(3) $$T_{e}=p_{n}\frac{L_{m}}{L_{r}}\left(i_{\mathrm{sq}}\psi_{\mathrm{rd}}-i_{\mathrm{sd}}\psi_{\mathrm{rq}}\right)$$

where *p_n_* is the pole-pair number.

In field orientation coordinate system, if the direction of the *d*-axis is along the direction of rotor flux *ψ_r_*, then

(4) $$\left\{\begin{array}{c} \psi_{\mathrm{rd}}=\psi_{r}\\ \psi_{\mathrm{rq}}=0 \end{array}\right.$$

The electromagnetic torque can be simplified as

(5) $$T_{e}=p_{n}\frac{L_{m}}{L_{r}}i_{\mathrm{sq}}\psi_{r}$$

The motion equation of a three-phase induction motors is given as

(6) $$T_{e}=T_{L}+J\frac{1}{p_{n}}\frac{d\omega }{dt}$$

where *T_L_* is the load torque and *J* is the rotary inertia.

**Figure 1:**
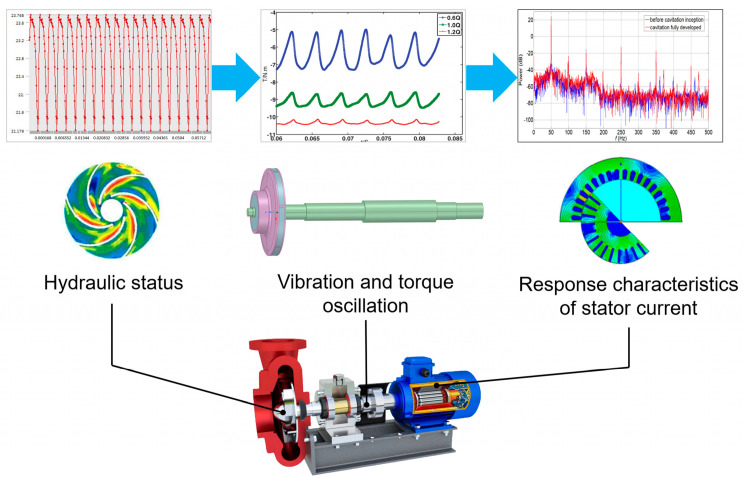
Hydro−mechanical−electric coupling in centrifugal pumps.

Based on Equations (5) and (6), it can be derived that the torque oscillations applied in *T_L_* would be transmitted to *T_e_* and reflected in *i_sq_*. This is the basic principle used to diagnose the operation condition of centrifugal pumps based on the stator current signals. The theoretical basis can be described as the hydro-mechanical-electric coupling effect in centrifugal pumps, as illustrated in Figure 1. In [3], the coupling effect was validated using Matlab/Fluent joint simulation, which indicates that the operation conditions of centrifugal pumps are possibly reflected in stator current signals.

**Figure 2:**
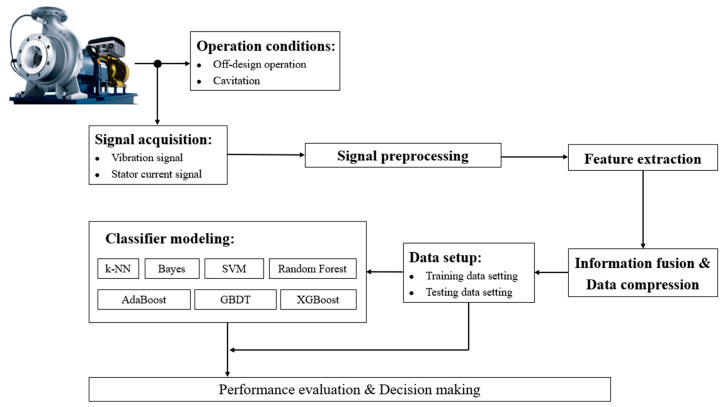
Operation diagnosis methodology for centrifugal pumps based on vibration and stator current analyses.

The procedure to diagnose the operation condition for centrifugal pumps is shown in Figure 2. Vibration and stator current signals are recorded simultaneously in a centrifugal pump under healthy, off-design conditions and different levels of cavitation. Signal preprocessing algorithms are performed to extract suitable features. The fusion of vibration and stator current information and data compression are considered necessary parts of the diagnosis method. After completing the above steps, different classifier models are implemented and evaluated based on their performances.

## 3. Signal Preprocessing and Feature Extraction

### 3.1. Signal Preprocessing and Feature Extraction of the Vibration Signals

The raw vibration signals have strong non-stationary randomness, which is not generally informative [29]. The wavelet threshold function is a widely used signal processing method for noise reduction and signal reconstruction. When using wavelet methods to decompose noisy signals, as the decomposition scale increases, the noise component tends to concentrate on relatively small wavelet coefficients. The basic principle of the wavelet threshold function is to find an appropriate noise threshold to suppress the signal coefficients below the threshold and retain the signal coefficient above the threshold. The most common threshold functions are the hard threshold function and the soft threshold function, which are defined as

(7) $$y_{j,k}=\left\{\begin{array}{cc} \omega_{j,k}, & \left|\omega_{j,k}\right|>\lambda \;\\ 0, & \left|\omega_{j,k}\right|\leq \lambda \end{array}\right.$$

(8) $$y_{j,k}=\left\{\begin{array}{cc} \mathrm{sgn}\left(\omega_{j,k}\right)\times \left(\left|\omega_{j,k}\right|-\lambda \right), & \left|\omega_{j,k}\right|>\lambda \;\\ 0, & \left|\omega_{j,k}\right|\leq \lambda \end{array}\right.$$

The hard thresholding function can preserve the basic characteristics of the original signals. However, the hard thresholding function is discontinuous at *ω_j,k_* = ±*λ*, which could cause signal oscillations in the reconstructed signals. In contrast, the soft thresholding function has the advantage of being continuous at *ω_j,k_* = ±*λ*, but there exists a difference between *ω_j,k_* and *y_j,k_*. This would cause the loss of useful information in the signals. Therefore, an improved wavelet threshold function is introduced in this paper for vibration signal denoising. 

The improved wavelet function is given by

(9) $$y_{j,k}=\left\{\begin{array}{cc} \mathrm{sgn}\left(\omega_{j,k}\right)\times \left(\left|\omega_{j,k}\right|-(\lambda /\alpha )\times \beta^{\sqrt{\omega_{j,k}^{2}-\lambda^{2}}}\right), & \left|\omega_{j,k}\right|>\lambda \;\\ \mathrm{sgn}\left(\omega_{j,k}\right)\times (\alpha -1/\alpha )\times \exp\left(10\times \left(\left|\omega_{j,k}\right|-\lambda \right)\right)\times \left|\omega_{j,k}\right|, & \left|\omega_{j,k}\right|\leq \lambda \end{array}\right.$$

where *α* and *β* are the two tuning parameters. These two parameters can be changed to adapt to different noise intensities. 

**Figure 3:**
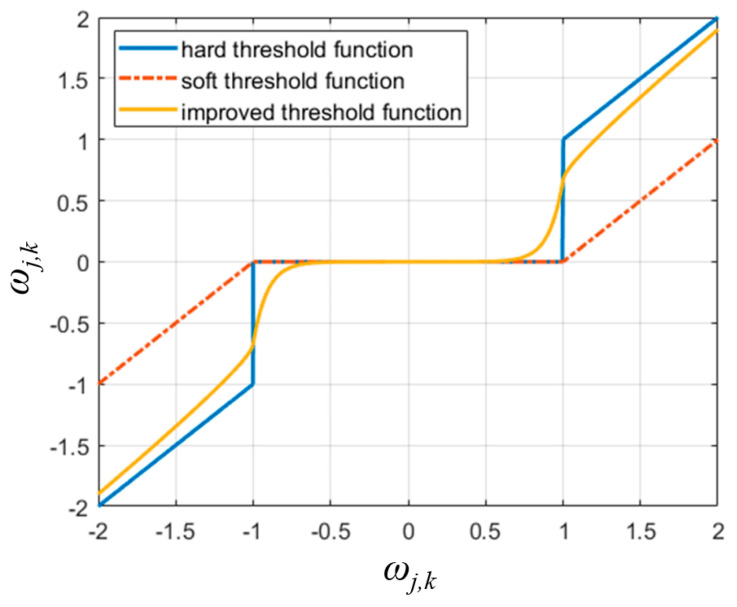
The improved wavelet threshold function compared with the hard threshold function and the soft threshold function.

The improved wavelet threshold function curve (with α = 2, *β* = 0.5) compared with the curves of the hard threshold function and the soft threshold function is shown in Figure 3.

From Equation (9), it can be derived that $\underset{\omega_{j,k}\to \lambda^{+}}{\lim}=\underset{\omega_{j,k}\to \lambda^{-}}{\lim}=\left(\alpha -1/\alpha \right)\times \lambda$. Meanwhile, if *β* ∈ (0, 1), it can be derived that $\underset{\omega_{j,k}\to \infty }{\lim}y_{j,k}=\omega_{j,k}$. This means that the improved threshold function has the advantages of both the hard threshold function and the soft threshold function.

The adaptive threshold *δ* is determined using the method introduced in [30]:

(10) $$\delta =\sigma {(2\ln(N))}^{\frac{1}{2}}$$

where *σ* is the noise standard deviation and *N* is the length of the signals.

After conducting the wavelet threshold function for vibration preprocessing, the Pearson correlation-based feature selection strategy introduced in [31] is used to select input parameters for the classifiers. The following dimensional and non-dimensional parameters in both time and frequency domains are considered: (1) root mean square, (2) standard deviation, (3) waveform factor, (4) kurtosis, (5) peak factor, (6) impulse factor, (7) clearance factor, (8) frequency center, and (9) root-mean-square frequency.

Meanwhile, aiming to measure the signal uncertainty changing with the operation condition, the following parameters based on the concept of information entropy are also adopted as operation indicators for centrifugal pumps as follows:

(10) Power spectral entropy:

The power spectral entropy provides a quantitative description of the complexity of signals’ energy distribution in the frequency domain. For a discrete-time signal sequence $\left\{\left.x(n)\right|n=0,1,\ldots ,\;N\;-1\right\}$, according to the Parseval theorem

(11) $$S(n)=\frac{1}{N}{\left|\mathrm{DFT}\left[x_{n}\right]\right|}^{2}$$

(12) $$\sum_{k=0}^{N-1}{\left|x_{n}\right|}^{2}=\sum_{k=0}^{N-1}{\left|S(k)\right|}^{2}$$

where DFT[*x_n_*] is the discrete Fourier transform of the original signals. 

The power spectral density entropy psdE is defined as

(13) $$\mathrm{psdE}=-\sum_{k=0}^{N-1}p_{k}\log p_{k}\;p_{k}=\frac{S(k)}{\sum_{k=0}^{N-1}S(k)}$$

where *p_k_* represents the ratio of the energy of the *k*th spectral line to the total spectral energy.

(11) VMD energy entropy:

The concept of mode decomposition energy entropy was first proposed in [32] for roller bearing fault diagnosis. In this research, the energy entropy is obtained based on the VMD results. Variational mode decomposition (VMD) is an effective way to decompose signals into a discrete number of sub-signals that have specific sparsity properties while reproducing the input [33]. It is reported that VMD is almost free from mode mixing difficulties [34,35]. In VMD, each mode *k* is assumed to be the most compact around a center pulsation *ω_k_*. The constrained variational problem can be described as

(14) $$\begin{array}{c} \min\\ \left\{u_{k}\right\},\left\{\omega_{k}\right\} \end{array}\left\{\sum_{k}\left\|\partial_{t}\left[\left(\delta_{t}+\frac{j}{\pi t}\right)*u_{k}\left(t\right)\right]\exp(-j\omega_{k}t)\right\|\begin{array}{c} 2\\ 2 \end{array}\right\}\;s.t.\;\sum_{k}u_{k}=f$$

where *f* is the real-valued input signals, *u_k_* are the sub-signals, *ω_k_* are the center frequencies of the sub-signals, and *δ* indicates the Dirac distribution subjected to $\sum u_{k}(t)=f(t)$.

The Lagrangian multiplier *λ* and penalty factor *α* are introduced to transform the constrained variational problem into an unconstrained one [34]. Once the VMD is implemented, vibration signals can be decomposed into n sub-signals and a residue component

(15) $$S(t)=\sum_{k=1}^{n}{\mathrm{IMF}}_{k}+r_{n}$$

The VMD energy entropy is defined as

(16) $$\;\mathrm{VMD}\;\mathrm{eE}=-\sum_{k=1}^{n}P_{k}\log P_{k}$$

where *P_k_* = *E_k_*/*E* is the ratio of the energy of IMF*_k_* to the total signal energy.

(12) Fuzzy entropy:

The definition of fuzzy entropy was first proposed in [35] for EMG signal processing. Fuzzy entropy was also used in the diagnosis of rotating machines in [36]. For a given time sequence *x*(*i*), the vector set sequences are constructed by

(17) $$X^{m}\left(i\right)=\left[x\left(i\right),x\left(i+1\right),\ldots ,x\left(i+m-1\right)\right]-x_{0}\left(i\right),\;i=1,\;2,\;\ldots ,\;N$$

(18) $$x_{0}\left(i\right)=\frac{1}{m}\sum_{k=0}^{m-1}x(i+k)$$

For a certain vector sequence *X*(*i*), the distance between *X^m^*(*i*) and *X^m^*(*j*) is defined as the maximum value of their corresponding scalar points as

(19) $$d_{\mathrm{ij}}^{m}=\begin{array}{c} \max\\ k\in (0,m-1) \end{array}\left|\left(x(i+k)-x_{0}(i)\right)-\left(x\left(j+k\right)-x_{0}(j)\right)\right|$$

A fuzzy function is introduced to measure the similarity between *X*(*i*) and *X*(*j*). Given *n* and *r*, the fuzzy function is given by

(20) $$D_{\mathrm{ij}}^{m}=\exp({\left(-(d_{\mathrm{ij}}^{m}\right)}^{n}/r))$$

The function $\phi^{m}$ is defined as

(21) $$\phi^{m}(n,r)=\frac{1}{N-m}\sum_{i=1}^{N-m}\left(\frac{1}{N-m-1}\sum_{j=1,j\neq i}^{N-m}D_{\mathrm{ij}}^{m}\right)$$

Similarly, *ϕ*^m+1^ can be obtained as

(22) $$\phi^{m+1}(n,r)=\frac{1}{N-m}\sum_{i=1}^{N-m}\left(\frac{1}{N-m-1}\sum_{j=1,j\neq i}^{N-m}D_{\mathrm{ij}}^{m+1}\right)$$

The fuzzy entropy of a time signal sequence with the finite data length *N* is defined by

(23) $$\mathrm{FuzzyEn}\left(m,\;n,\;r,\;N\right)=\ln\phi^{m}\left(n,r\right)-\ln\phi^{m+1}\left(n,r\right)$$

where *m* is the embed dimension, *n* determines the gradient of the fuzzy function boundary, and *r* determines the width of the fuzzy function boundary. In this research, *m*, *n*, and *r* are fixed as 2, 2, and 0.15, respectively.

### 3.2. Signal Preprocessing and Feature Extraction of the Current Signals

Rotor rotational speed frequency and blade passing frequency are inherent in centrifugal pumps, which indicate the torque oscillation characteristics, given by

(24) $$f_{r}=2\left(1-s\right)f_{e}/P_{n}$$

(25) $$f_{\mathrm{BPF}}=k_{b}f_{r}$$

where *s*, *f_e_*, *P_n_*, and *k_b_* are the slip, the power frequency, the pole-pair number, and the blade number. 

For the stator current signals of centrifugal pumps, *f_r_* and its integer multiples (typically *f*_BPF_) would produce observable harmonics due to power frequency modulation as

(26) $$f_{s}=\left|f_{e}\pm Nf_{r}\right|$$

where *N* is a positive integer.

However, the current features are easily affected by the changes in the power supply. The amplitudes of the characteristic harmonics may change due to the power frequency fluctuations regardless of the pump conditions and induce false alarms. To address this issue, the Park vector modulus transformation is applied to process the original stator current signals. Currents in the *d-q* coordinate system [*i_d_*, *i_q_*] can be obtained as

(27) $$\left[\begin{array}{c} i_{d}\\ i_{q} \end{array}\right]=\sqrt{\frac{2}{3}}\left[\begin{array}{ccc} 1 & -\frac{1}{2} & -\frac{1}{2}\\ 0 & \frac{\sqrt{3}}{2} & \frac{\sqrt{3}}{2} \end{array}\right]\left[\begin{array}{c} i_{A}\\ i_{B}\\ i_{C} \end{array}\right]$$

where *i_A_*, *i_B_* and *i_C_* are the three-phase stator currents.

In real industrial applications, due to the non-linear loads, the magnitudes of three-phase stator currents may not be perfectly balanced. Suppose *I_A_* = *I_m_*sin(2$\pi f_{e}t$), and the difference between the magnitude of *I_A_* and the magnitude of *I_C_* is *k*, then the Park vector $I_{S}(t)\;$ and the square of the Park vector modulus of the stator current ${\left|I_{S}(t)\right|}^{2}$ can be expressed as [37]:

(28) $$I_{S}(t)=\sqrt{\frac{2}{3}\left[i_{A}\left(t\right)+i_{B}\left(t\right)e^{j(2\pi /3)}+i_{C}\left(t\right)e^{-j(2\pi /3)}\right]}$$

(29) $${\left|I_{S}\left(t\right)\right|}^{2}=\left(\frac{3}{2}-\left(1-k\right)\frac{k+2}{3}\right)I_{m}^{2}\;-\left(1-k\right)I_{m}^{2}\left[\frac{1-k}{3}\cos(4\pi f_{e}t-\frac{2}{3}\pi )+\frac{1}{2}\cos\left(4\pi f_{e}t+\frac{2}{3}\pi \right)+\frac{\sqrt{3}}{2}\sin\left(4\pi f_{e}t+\frac{2}{3}\pi \right)\right]$$

Under an ideal condition where the three-phase stator currents are perfectly balanced (*k* = 1), the ${\left|I_{S}\left(t\right)\right|}^{2}$ contains no oscillation component. In a centrifugal pump system, the hydraulic torque oscillations would make the stator current waveforms distorted.

For an induction motor-centrifugal pump system with torque oscillations related to the operation conditions, its three-phase stator currents can be expressed as

(30) $$i_{A}\left(t\right)=I_{m}\cos\left(2\pi f_{e}t\right)+I_{l1}\cos\left[2\pi \left(f_{e}-f_{r}\right)t-\varphi_{l1}\right]\;+I_{r1}\cos\left[2\pi \left(f_{e}+f_{r}\right)t-\varphi_{r1}\right]+I_{l2}\cos[2\pi (Nf_{r}-f_{e})t-\varphi_{l2}]\;+I_{r2}\cos[2\pi (Nf_{r}+f_{e})t-\varphi_{r2}]\;i_{B}\left(t\right)=I_{m}\cos\left(2\pi f_{e}t-\frac{2}{3}\pi \right)+I_{l1}\cos\left[2\pi \left(f_{e}-f_{r}\right)t-\varphi_{l1}-\frac{2}{3}\pi \right]\;+I_{r1}\cos\left[2\pi \left(f_{e}+f_{r}\right)t-\varphi_{r1}-\frac{2}{3}\pi \right]+I_{l2}\cos\left[2\pi \left(Nf_{r}-f_{e}\right)t-\varphi_{l2}-\frac{2}{3}\pi \right]\;+I_{r2}\cos\left[2\pi \left(Nf_{r}+f_{e}\right)t-\varphi_{r2}-\frac{2}{3}\pi \right]\;i_{C}\left(t\right)=I_{m}\cos\left(2\pi f_{e}t+\frac{2}{3}\pi \right)+I_{l1}\cos\left[2\pi \left(f_{e}-f_{r}\right)t-\varphi_{l1}+\frac{2}{3}\pi \right]\;+I_{r1}\cos\left[2\pi \left(f_{e}+f_{r}\right)t-\varphi_{r1}+\frac{2}{3}\pi \right]+I_{l2}\cos\left[2\pi \left(Nf_{r}-f_{e}\right)t-\varphi_{l2}+\frac{2}{3}\pi \right]\;+I_{r2}\cos\left[2\pi \left(Nf_{r}+f_{e}\right)t-\varphi_{r2}+\frac{2}{3}\pi \right]$$

where *I_m_* is the amplitude of the power frequency component. *I_l_*_1_, *I_r_*_1_, *I_l_*_2_, and *I_r_*_2_ are the amplitudes of the characteristic sidebands. *φ_l_*_1_, *φ_r_*_1_, *φ_l_*_2_, and *φ_r_*_2_ are the initial phase angles corresponding to the characteristic sidebands.

The square of the Park vector modulus of the stator current can be obtained by

(31) $${\left|I_{S}\left(t\right)\right|}^{2}=\frac{3}{2}\left(I_{m}^{2}+I_{l1}^{2}+I_{l2}^{2}+I_{r1}^{2}+I_{r2}^{2}\right)+3I_{m}I_{l1}\cos\left[2\pi f_{r}t-\varphi_{l1}\right]+3I_{m}I_{r1}\cos\left[2\pi f_{r}t-\varphi_{r1}\right]\;+3I_{m}I_{l2}\cos\left[2\pi (Nf_{r})t+\varphi_{l2}\right]+3I_{m}I_{r2}\cos\left[2\pi (Nf_{r})t-\varphi_{r2}\right]\;+3I_{l1}I_{r1}\cos\left[2\pi {(2f}_{r})t-\varphi_{l1}-\varphi_{r1}\right]+3I_{r1}I_{r2}\cos\left[2\pi (N-1)f_{r}t+\varphi_{r1}-\varphi_{r2}\right]\;+3I_{l1}I_{r2}\cos\left[2\pi \left(N+1\right)f_{r}t-\varphi_{l1}-\varphi_{r2}\right]+3I_{l2}I_{r1}\cos[2\pi (N+1)f_{r}t+\varphi_{l2}-\varphi_{r1}]\;+3I_{l1}I_{l2}\cos[2\pi \left(N-1\right)f_{r}t+\varphi_{l1}+\varphi_{l2}]+3I_{l2}I_{r2}\cos\left[2\pi \left(2N\right)f_{r}t+\varphi_{l2}-\varphi_{r2}\right]$$

where 3/2·(*I_m_*_2_ + *I_l_*_1_^2^ + *I_l_*_2_^2^ + *I_r_*_1_^2^ + *I_r_*_2_^2^) is the DC offset of the square of the Park vector modulus considering *I_l_*_1_, *I*_r1_, *I*_l2_ and *I*_r2_ are far less than *I_m_*. The square of the Park vector modulus can be further simplified as

(32) $${\left|I_{S}\left(t\right)\right|}_{\mathrm{sim}}^{2}=\frac{{\left|I_{S}\left(t\right)\right|}^{2}-{\left|I_{S}\left(t\right)\right|}_{\mathrm{DC}}^{2}}{I_{m}}=3I_{l1}\cos\left[2\pi f_{r}t-\varphi_{l1}\right]+3I_{r1}\cos\left[2\pi f_{r}t-\varphi_{r1}\right]\;+3I_{l2}\cos[2\pi (Nf_{r})t+\varphi_{l2}]+3I_{r2}\cos[2\pi (Nf_{r})t-\varphi_{r2}]$$

It can be seen from Equation (32) that the power frequency component is demodulated in ${\left|I_{S}\left(t\right)\right|}_{\mathrm{sim}}^{2}$. The characteristic sidebands corresponding to *f_r_* and *Nf_r_* can be used to reflect the intensity of torque oscillations. These sidebands extracted from the spectrum of ${\left|I_{S}\left(t\right)\right|}_{\mathrm{sim}}^{2}$ are immune to power frequency interference and can be used as the stator current indicators used for pump diagnosis.

Meanwhile, it should be noted that when pumps operate under cavitation conditions, high-frequency fluid-borne noise is created by the subsequent collapse of bubbles, and low-frequency pulsations are created through large fluctuations of the cavitation zones [1]. Both sources could cause broadband oscillations in pumps’ torque, which would be reflected as sideband noise around the characteristic harmonics and high-frequency noise of the stator current.

Therefore, the harmonic indicator alone may not be capable of detecting cavitation. In this research,${\left|I_{S}\left(t\right)\right|}_{\mathrm{sim}}^{2}$ is also decomposed by the VMD method to analyze the energy distribution across different frequency bands, and an indicator that reflects the energy ratio of each IMF component is developed as

(33) $$\mathrm{Energy}\;\mathrm{Ratio}=\frac{{u_{k}\left(t\right)}^{2}}{{\left({\left|I_{S}\left(t\right)\right|}_{\mathrm{sim}}^{2}\right)}^{2}}\;(k=1,\;2,\;3\ldots \;n)$$

where $u_{k}\left(t\right)$ are the IMF components of ${\left|I_{S}\left(t\right)\right|}_{\mathrm{sim}}^{2}$.

Meanwhile, to obtain the energy distribution of the stator current signal in a sense of probability, the Hilbert marginal spectrum is also employed for feature extraction. The Hilbert transform is applied to each IMF component as

(34) $$H[u_{k}(t)]=\frac{1}{\pi }\int_{-\infty }^{\infty }\frac{u_{k}(\tau )}{t-\tau }d\tau$$

The analytic signals can be constructed by

(35) $$z(t)=u_{k}(t)+\mathrm{jH}[u_{k}(t)]=A_{k}(t)\exp(j\theta_{k}(t))$$

where $A_{k}(t)=\sqrt{{\left(u_{k}(t)\right)}^{2}+{\left\{H\left[u_{k}(t)\right]\right\}}^{2}}$ is the instantaneous amplitude and $\theta_{k}(t)=\arctan\{H\left[u_{k}(t)\right]/u_{k}(t)\}$ is the instantaneous phase. The instantaneous frequency $f_{k}(t)\;$ can be calculated as

(36) $$f_{k}(t)=\frac{d\theta_{k}\left(t\right)}{2\pi \mathrm{dt}}$$

The Hilbert spectrum describes the changing rule of signals over time and frequency, which is given by

(37) Hf, t=Re∑i=1nAk(t)exp(j2π∫fk(t)dt)

The Hilbert marginal spectrum can be obtained based on the integration of the Hilbert spectrum

(38) $$h(f)=\int_{0}^{T}H(f,t)dt$$

The Hilbert marginal spectrum provides the local feature accuracy of signals. In this research, the energy of the characteristic frequency band centered at *f_r_* and *f*_BPF_ in the Hilbert marginal spectrum is also used as a current-based indicator, in order to capture local detail fluctuations of ${\left|I_{S}\left(t\right)\right|}_{\mathrm{sim}}^{2}$ caused by cavitation-induced torque oscillations.

## 4. Intelligent Diagnosis Algorithms

In this research, t-Distributed Stochastic Neighbor Embedding (t-SNE) is used for vibration–current fusion and data compression. It works by modeling the similarity between pairs of high-dimensional data points as a probability distribution and then finding a low-dimensional representation of the data that minimizes the divergence between the high-dimensional and low-dimensional distributions. In this algorithm, the conditional probability that represents the similarity between *x_i_* and *x_j_* is given by

(39) $$P_{j\left|i\right.}=\frac{\exp\left(-{\left\|x_{i}-x_{j}\right\|}^{2}/2\sigma_{i}^{2}\right)}{\sum_{k\neq i}\exp\left(-{\left\|x_{i}-x_{k}\right\|}^{2}/2\sigma_{i}^{2}\right)}$$

where *σ_i_* is the variance of the Gaussian that is centered on *x_i_*.

The joint probabilities *p_ij_* in the high-dimensional space is calculated by symmetrizing the conditional probabilities

(40) $$P_{\mathrm{ij}}=\frac{P_{j\left|i\right.}+P_{i\left|j\right.}}{2n}$$

t-SNE aims to minimize the Kullback–Leibler divergence between the Gaussian distribution of the data points in the original high-dimensional space and the Student’s t-distribution of the data points in the lower-dimensional space. The joint probabilities in the low-dimensional space can be calculated as

(41) $$q_{\mathrm{ij}}=\frac{{\left(1+{\left\|y_{i}-y_{j}\right\|}^{2}\right)}^{-1}}{\sum_{k\neq l}{\left(1+{\left\|y_{k}-y_{l}\right\|}^{2}\right)}^{-1}}$$

The Kullback–Leibler divergence between the two joint probability distributions *P* and *Q* can be calculated as

(42) $$C=KL(P\left\|Q\right.)=\sum_{i}\sum_{j}p_{\mathrm{ij}}\log p_{\mathrm{ij}}-p_{\mathrm{ij}}\log q_{\mathrm{ij}}$$

To minimize the Kullback–Leibler divergence, the optimized gradient can be calculated as

(43) $$\frac{\partial C}{\partial y_{i}}=4\sum_{j}\left(p_{\mathrm{ij}}-q_{\mathrm{ij}}\right)\left(y_{i}-y_{j}\right){\left(1+{\left\|y_{k}-y_{l}\right\|}^{2}\right)}^{-1}$$

The embedded points in the low-dimensional space at the *t*th iteration can be updated as

(44) $$y^{\left(t\right)}=y^{(t-1)}+\gamma \frac{\partial C}{\partial y}+\alpha (t)(y^{(t-1)}-y^{(t-2)})$$

where γ is the learning rate and α(t) is the momentum at the *t*th iteration.

In this research, several classification algorithms used to perform operation diagnosis are evaluated, namely: 1. Mahalanobis distance-based k-nearest neighbor (MD-KNN); 2. Euclidean distance-based k-nearest neighbor (ED-KNN); 3. Bayes learning; 4. support vector machines; 5. random forest; 6. Adaboost; 7. gradient boosting decision tree (GBDT); and 8. eXtreme Gradient Boosting (XGBoost).

## 5. Experimental Section

### 5.1. Experimental Setup

**Figure 4:**
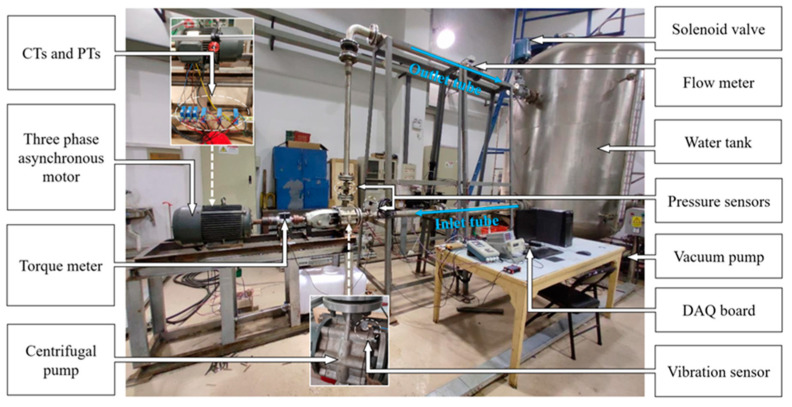
The experimental test bench.

A comprehensive experiment test bench was designed to automatically detect off-design operation and cavitation for the centrifugal pump, as illustrated in Figure 4. An IS65-50-160-00 centrifugal pump was tested. The pump casing was made of stainless steel and the impeller was made of cast iron. The drive induction motor was connected to a 380 V, 50 Hz AC power supply. The parameters of the centrifugal pump and induction motor are shown in Table 1.

As depicted, the fluid loop mainly consisted of the centrifugal pump, the inlet tube, the outlet tube, and the water tank. The solenoid valve, coupled to a functional analog voltage generator, was installed in the outlet tube to obtain different flow rates corresponding to the valve opening. The vacuum pump, linked to the water tank, was used to reduce the inlet pressure and enable the centrifugal pump to operate under cavitation conditions.

### 5.2. Sensors Used in the Experiment

In this experiment, a torque meter, an electromagnetic flowmeter, and two pressure sensors mounted on the pump inlet and pump outlet were used to monitor the pump’s performance parameters. Three-phase stator current signals were measured by three Hall-effect current sensors. A triaxial accelerometer was used to record vibration signals in the radial, longitudinal, and axial directions. An NI-USB-6343 base acquisition module was deployed for data acquisition. The vibration and current signals were measured simultaneously with a sampling frequency of 10 kHz under stable-state operation conditions. The main parameters of the sensors used in the experiment are shown in Table 2.

### 5.3. Experiment Process

The centrifugal pump was tested first at 0*Q_des_*, 0.1*Q_des_*, 0.2*Q_des_*, 0.3*Q_des_*, 0.4*Q_des_*, 0.5*Q_des_*, 0.6*Q_des_*, 0.7*Q_des_*, 0.8*Q_des_*, 0.9*Q_des_*, 1.0*Q_des_*, 1.1*Q_des_*, 1.2*Q_des_*, and 1.3*Q_des_*. Vibration and current signals under off-design operation conditions were obtained, as well as the *Q*-*H*, *Q*-*η*, and *Q*-*P* characteristic curves. According to [1], the preferred operation range for a centrifugal pump is defined as 0.85 < q* < 1.1, which is set as the health status for the tested centrifugal pump in this research. As for the cavitation experiments, drops in the heads of the suction impeller by 1% and 3% were determined as the two cavitation criteria, where a 1% drop in head indicated cavitation inception and a 3% drop in head represented the critical value at which cavitation was fully developed. Cavitation experiments were repeated at 0.8*Q_des_*, 1.0*Q_des_*, and 1.2*Q_des_*. The experiments were carried out for about 200 h for a period of 40 days.

## 6. Results and Discussion

**Figure 5:**
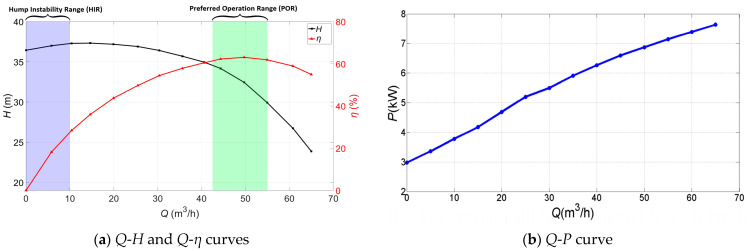
Characteristic curves of the centrifugal pump.

Figure 5 shows the *Q*-*H*, *Q*-*η*, and *Q*-*P* characteristic curves of the centrifugal pump. The centrifugal pump has the best efficiency at the design flow rate (50 m^3^/h). Meanwhile, the pump power increases with the increasing flow rate. As illustrated in Figure 5, the green shaded area depicts the preferred operation range (POR) of the tested centrifugal pump, and the purple shaded area depicts the hump instability range (HIR) of the tested centrifugal pump. The hump instability range is also called the positive slope at shut-off in [1]. As the flow rate gradually increases from the shut-off point, the head first gradually increases to the maximum value, and then it decreases as the flow rate increases. In this research, the HIR is defined as the operation range with ∂*H*/∂*Q* > 0 [1] (0–10 m^3^/h), which indicates that the increasing turbulent disturbance corresponds to the unstable characteristic range.

**Figure 6:**
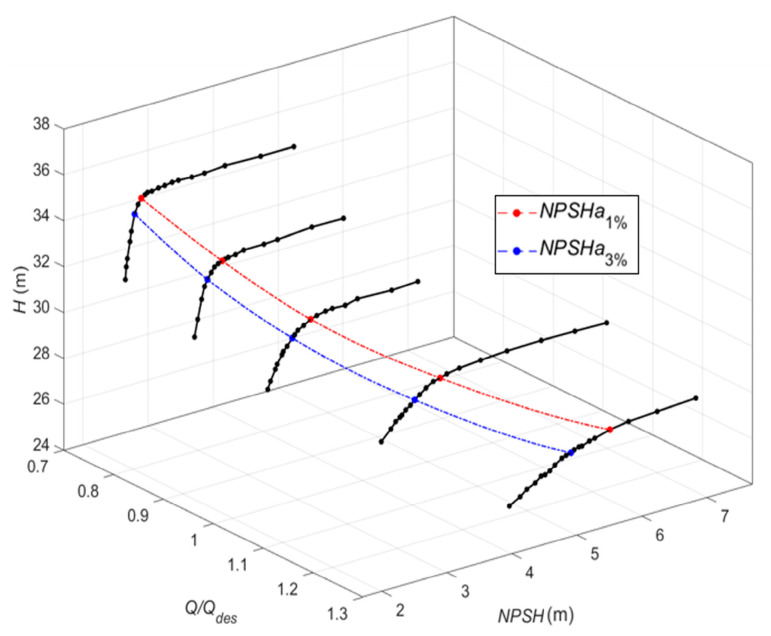
The cavitation curves of the centrifugal pump under different flowrates.

Figure 6 presents the pump cavitation characteristic curves at different flow rates. The smaller *NPSH* value means more serious cavitation. According to [1], the *NPSH*_1%_ is determined by a 1% drop in the total delivery head, indicating cavitation inception. The *NPSH*_3%_ is determined by a 3% drop in the total delivery head, indicating cavitation is fully developed.

### 6.1. Performance of the Vibration-Based Indicators

**Figure 7:**
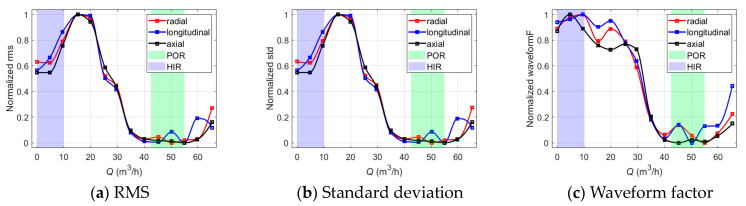

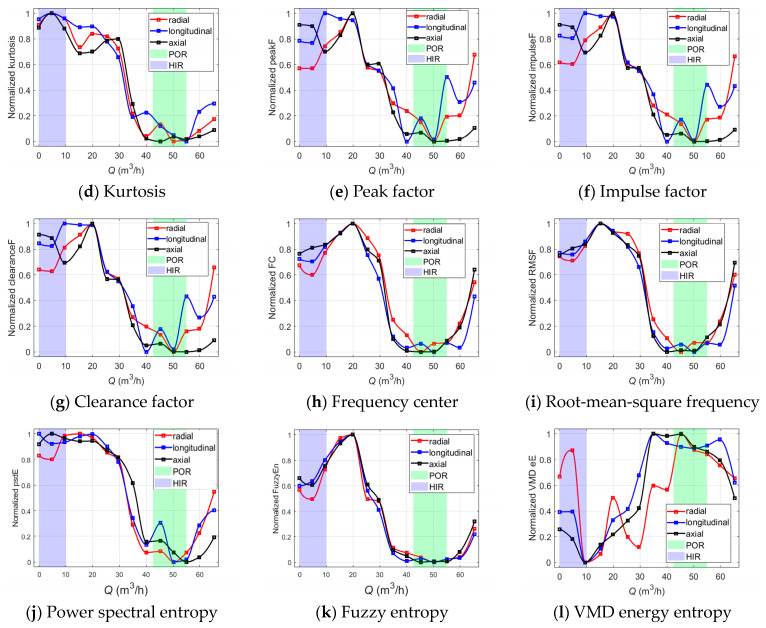
The vibration-based indicators versus *Q* for off-design operation detection.

Figure 7 shows the selected vibration-based indicators for the off-design operation diagnosis of the centrifugal pump. It can be seen that the indicator curves of the radial, longitudinal, and axial directions extracted from the denoised vibration signals show similar tendencies versus the flow rate.

The indicator curves (a)~(k) have significant saddle-type zones in the preferred operation range, and the indicator curve (l) has a significant hump-type zone in the preferred operation range. This means that these indicators have the ability to recognize the high-efficiency operation range of the centrifugal pump. Meanwhile, it is observed that the indicator curves (a)~(k) show upward trends near the shut-off point, which partially or totally overlap with the hump instability range. The indicators reach the extreme at 0.2~0.4q*, which is also the range where serious recirculation occurs at the impeller inlet and outlet. Once the indicator curves cross the instability range, they steadily fall with the increasing flow rate until they reach the preferred operation range. Moreover, it is observed that the VMD energy entropy presents the opposite tendency compared to (a)~(k). The VMD energy entropy basically decreases as the operation condition gets worse, which means the energy distributes mainly in the resonance frequency band, and the distribution uncertainty becomes relatively lower under the off-design operation conditions.

**Figure 8:**
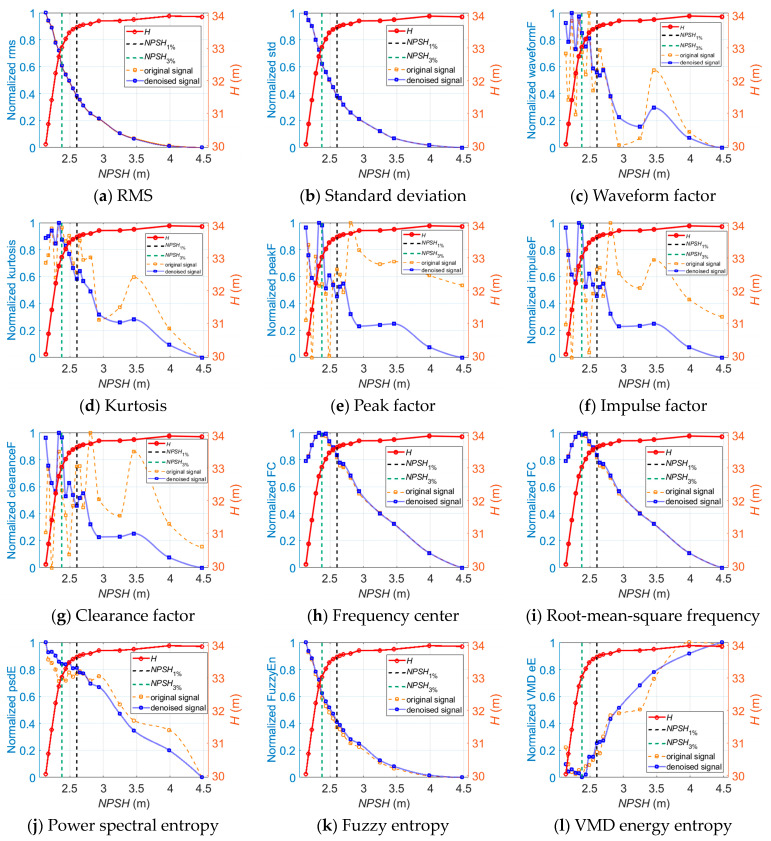
The longitudinal vibration-based indicators versus *NPSH* for cavitation detection at 1.0*Q_des_*.

As for cavitation diagnosis, Figure 8 illustrates the longitudinal vibration-based indicators versus *NPSH* at 1.0*Q_des_* before and after wavelet threshold denoising. It is observed that the indicators obtained from the denoised vibration signals show noticeably greater linear trends with the cavitation process than the original signal-based indicators.

**Figure 9:**
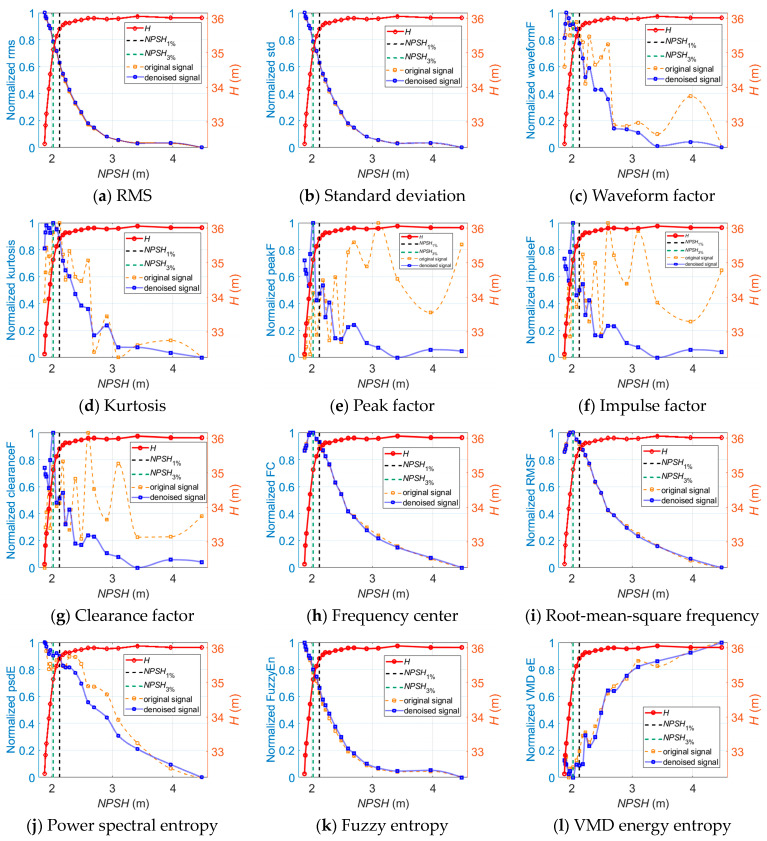
The longitudinal vibration-based indicators versus *NPSH* for cavitation detection at 0.8*Q_des_*.

**Figure 10:**
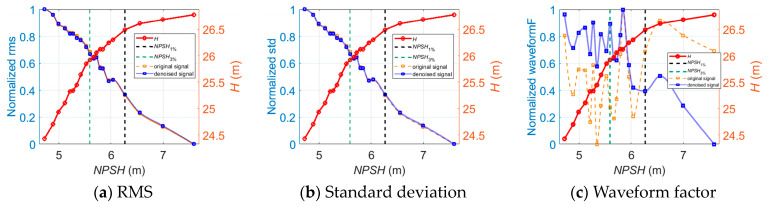

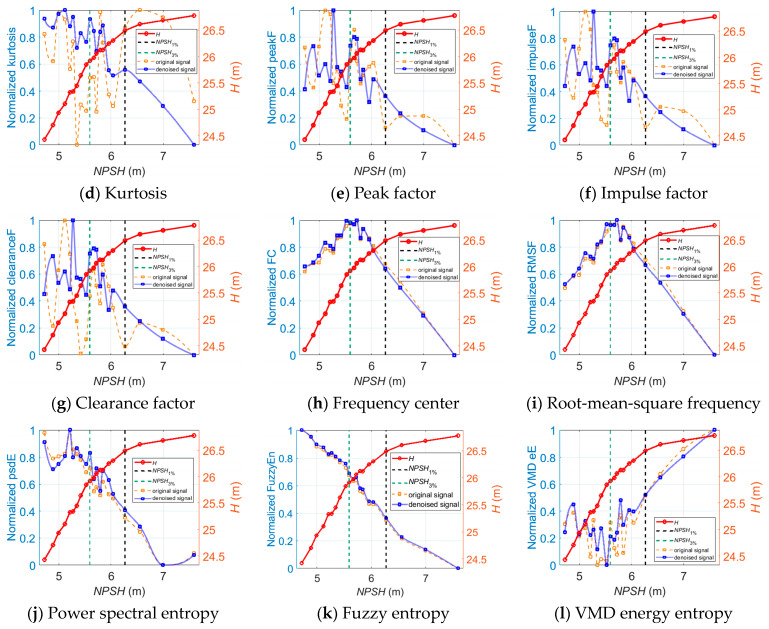
The longitudinal vibration-based indicators versus *NPSH* for cavitation detection at 1.2*Q_des_*.

Similar trends of the denoised longitudinal vibration-based indicators versus *NPSH* are also observed at 0.8*Q_des_* and 1.2*Q_des_*, as shown in Figure 9 and Figure 10. One thing that should be noted is that the inflection points of FC, RMSF, and VMD energy entropy appear near the *NPSH*_3%_ point at which cavitation is fully developed. The FC and RMSF indicators keep increasing as cavitation develops until they reach the critical value, and then they start to decrease. Meanwhile, the VMD energy entropy indicator exhibits an opposite trend. This is because during fully developed cavitation, bubbles collapse in close proximity to impeller walls and act like impulses. Vibration excited by pressure pulsations and resultant noise shift in the high-frequency direction, and the energy distribution of the vibration signals in each frequency band becomes uneven. The inflection points of FC, RMSF, and VMD energy entropy can be utilized to indicate the fully developed cavitation state in the centrifugal pump.

**Figure 11:**
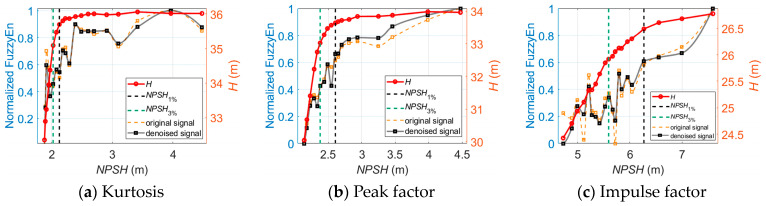
The axial vibration-based fuzzy entropy indicator versus *NPSH* for cavitation detection.

As for the analyses of the vibration signals in all three directions, the trends of the indicator curves are similar except for the fuzzy entropy indicator in the longitudinal and axial directions. Figure 11 shows the axial vibration-based fuzzy entropy indicator versus *NPSH* at 0.8*Q_des_*, 1.0*Q_des_*, and 1.2*Q_des_*.

As illustrated in Figure 11, the fuzzy entropy in the axial direction shows an opposite trend versus *NPSH* compared to that in the longitudinal direction. This is different from the detection of off-design operation—in this case, the vibration indicators in all three directions show similar trends versus *Q*. Therefore, an indicator that is only sensitive to cavitation in the centrifugal pump is developed as

(45) $${\mathrm{FuzzyEn}}_{\mathrm{combined}}=\frac{{\mathrm{FuzzyEn}}_{\mathrm{longitudinal}}}{{\mathrm{FuzzyEn}}_{\mathrm{axial}}}$$

Therefore, instead of using vibration indicators in all three directions, this research uses the twelve longitudinal vibration-based indicators and a combined indicator based on the fuzzy entropy in two directions to generate the vibration-based input matrix for the training of the classifiers.

### 6.2. Performance of the Current-Based Indicators

**Figure 12:**
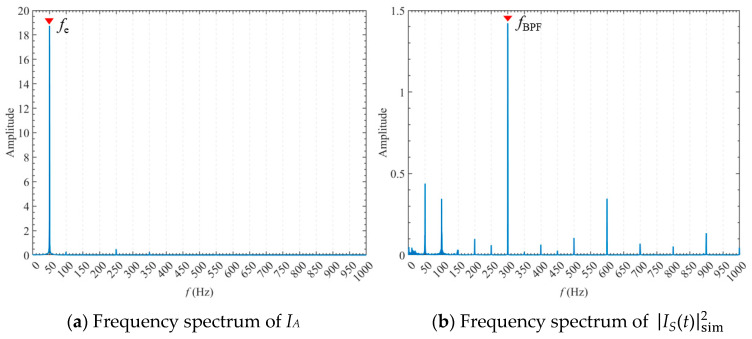
Frequency spectra of *I_A_* and ${\left|I_{S}(t)\right|}_{\mathrm{sim}}^{2}$.

Figure 12 shows the spectrum comparison between *I_A_* and ${\left|I_{S}\left(t\right)\right|}_{\mathrm{sim}}^{2}$. It can be seen that in the spectrum of *I_A_*, the major contribution comes from the power frequency component *f_e_* at approximately 50 Hz. The fundamental frequency component of ${\left|I_{S}\left(t\right)\right|}_{\mathrm{sim}}^{2}$ is the blade passing frequency *f*_BPF_, which represents the fundamental frequency of hydraulic torque oscillation of the centrifugal pump.

**Figure 13:**
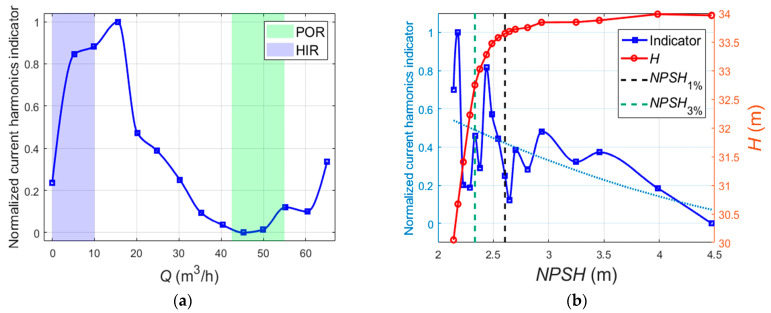
The stator current-based harmonic indicator for operation diagnosis of the centrifugal pump. (**a**) The stator current-based harmonic indicator versus *Q* for off-design operation detection, (**b**) The stator current-based harmonic indicator versus NPSH for cavitation detection at 1.0*Q_des_*.

According to Equation (33), the sum of the harmonic amplitudes of ${\left|I_{S}\left(t\right)\right|}_{\mathrm{sim}}^{2}$ is used as a stator current-based indicator for pump operation diagnosis. Figure 13a shows the stator current-based harmonic indicators versus *Q*. It can be seen that the changing trend of the harmonic indicators is similar to that of the vibration-based indicators, as shown in Figure 7a–k. The destabilizing effect on the pump characteristic in the hump instability range is reflected in the indicator curve as an increasing trend. Meanwhile, the harmonic indicator has its minimum in the preferred operation range, which represents the weakest hydraulic torque oscillations in a relatively stable state.

According to Equation (31), the sum of the harmonic amplitudes of ${\left|I_{S}\left(t\right)\right|}_{\mathrm{sim}}^{2}$ is used as a stator current-based indicator for pump operation diagnosis. Figure 13a shows the stator current-based harmonic indicators versus *Q*. It can be seen that the changing trend of the harmonic indicators is similar to that of the vibration-based indicators as shown in Figure 7a–k. The destabilizing effect on the pump characteristic in the hump instability range is reflected in the indicator curve as an increasing trend. Meanwhile, the harmonic indicator has its minimum in the preferred operation range, which represents the weakest hydraulic torque oscillations in a relatively stable state.

Figure 13b shows the measured total delivery head and the corresponding harmonics indicator versus *NPSH* with the pump set with cavitation at 1.0*Q_des_*. The indicator shows a fluctuating increasing trend as cavitation develops, as shown in the dotted trend line. However, the implosion of vapor bubbles in the pump appears chaotically. They can cause torque disturbances in a wide frequency range or at some specific frequency components; so, the cavitation may cause changes in the characteristic harmonics or energy of certain frequency bands in the stator current signals.

**Figure 14:**
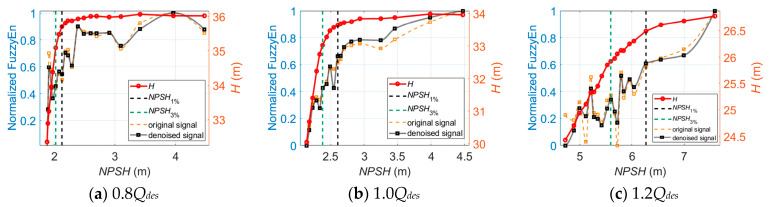
RMS of ${\left|I_{S}(t)\right|}^{2}$ versus *NPSH* for cavitation detection at different flow rates.

Figure 14 shows the RMS value of ${\left|I_{S}(t)\right|}^{2}\;$ versus *NPSH* at different flow rates. It is seen that the RMS indicator follows a trend similar to the cavitation characteristic curve. The RMS indicator reflects the trend of changes in the blade load of the centrifugal pump. As cavitation develops, the static pressure distribution on the surface of the blade is changed, which causes the decrease in blade load and is reflected in the decrease in the RMS of ${\left|I_{S}(t)\right|}^{2}$.

**Figure 15:**
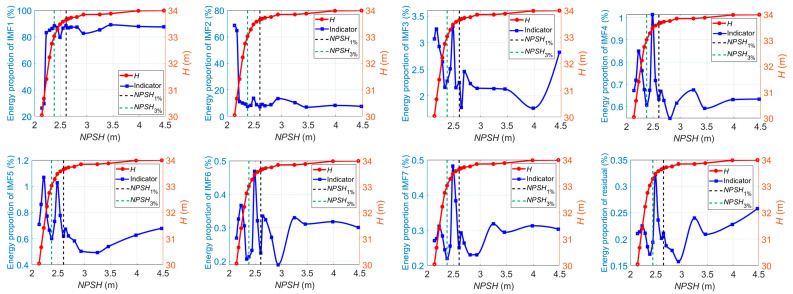
Energy ratios of IMF*_i_* versus *NPSH* for cavitation detection at 1.0*Q_des_*.

According to Equation (33), the energy ratios of IMF*_i_* to the whole-signal energy are also used as current-based indicators. Figure 15 shows the energy ratios of IMF*_i_* to the whole-signal energy versus the *NPSH* value at 1.0*Q_des_*, where the number of modes *K* = 8. IMF1, which contains the low-frequency component below 300 Hz, is the dominant signal source. As illustrated, the energy ratio of IMF1 fluctuates slightly as cavitation develops. When the critical value of *NPSH* is reached, the energy ratio of IMF1 begins to decrease steeply. Meanwhile, the energy ratios of other IMF components show a tendency to decrease at first; then, they increase, especially after cavitation is fully developed. The relative strength between the high-frequency component and the low-frequency component of the current signals could reflect the cavitation development process: at the beginning of cavitation, low-frequency pulsations within large amplitudes are created through large fluctuations of the cavitation zones. The compressibility of cavities may result in cavitation surges. These may excite the torsional vibration of shafting, which could cause low-frequency torque oscillations and be reflected in the current signals through the mechanical-electric coupling effect. At this stage, it can be seen that the energy proportion of the high-frequency current signal component keeps decreasing. As cavitation continues to develop, high-frequency fluid-borne noise and vibration are created by the implosion of vapor-filled zones (bubbles), acting like impulses to the metal itself and eroding it. The induced high-frequency torque oscillations could be reflected in the stator current signal. As a result, it is observed that the high-frequency energy ratio of the current signal increases, and, on the contrary, the low-frequency energy ratio falls steeply.

**Figure 16:**
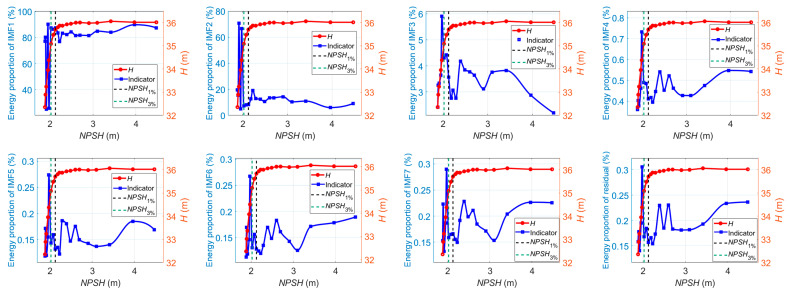
Energy ratios of IMF*_i_* versus *NPSH* for cavitation detection at 0.8*Q_des_*.

**Figure 17:**
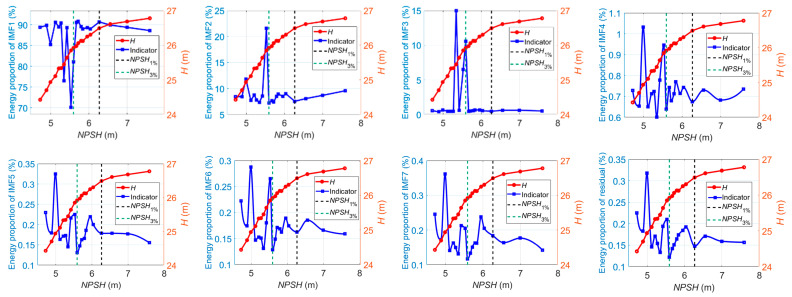
Energy ratios of IMF*_i_* versus *NPSH* for cavitation detection at 1.2*Q_des_*.

Similar patterns of variation in the energy ratios of IMF*_i_* also occur at 0.8*Q_des_* and 1.2*Q_des_*, as shown in Figure 16 and Figure 17.

**Figure 18:**
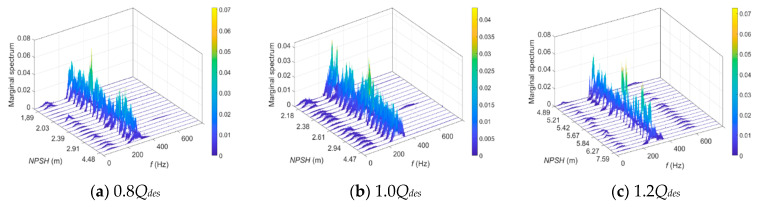
Marginal spectra of ${\left|I_{S}\left(t\right)\right|}_{\mathrm{sim}}^{2}$ for different *NPSH* values at different flow rates.

The marginal spectra of ${\left|I_{S}\left(t\right)\right|}_{\mathrm{sim}}^{2}$ for different *NPSH* values at different flow rates are shown in Figure 18. It is observed that the dominant signal component is mostly concentrated in the frequency band centered at 300 Hz with a bandwidth of about 100 Hz. Broadband components centered at 2 × RF with a bandwidth of about 100 Hz are also noticed in the spectra. These two broadband components are the reflection of pump hydraulic torque oscillations. The energy of each frequency band can be calculated by amplitude accumulation. The energy ratio of the frequency bands centered at BPF to the frequency bands centered at 2 × RF is used as the marginal spectrum indicator.

**Figure 19:**
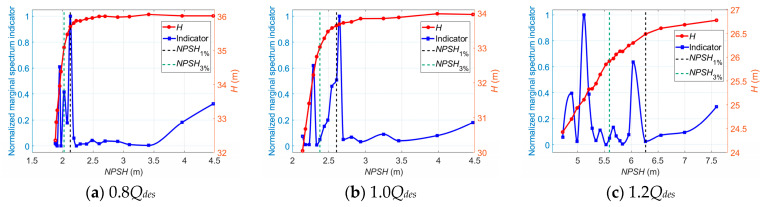
Marginal spectrum indicator versus *NPSH* for cavitation detection at different flow rates.

Figure 19 shows the normalized marginal spectrum indicator versus the *NPSH* value at different flow rates.

As illustrated, the indicator value is maintained at a relatively low level, with a slightly downward trend before the onset of cavitation. When cavitation occurs in the pump, a steep increase followed by a strong fluctuation occurs, which means there is a strong energy transfer from RF to BPF in hydraulic torque oscillations, which could be subject to the effect of cavitation.

Conclusively, these stator current-based indicators are used to generate the current-based input matrix for training the classifiers.

### 6.3. Performance of the Classifiers

**Figure 20:**
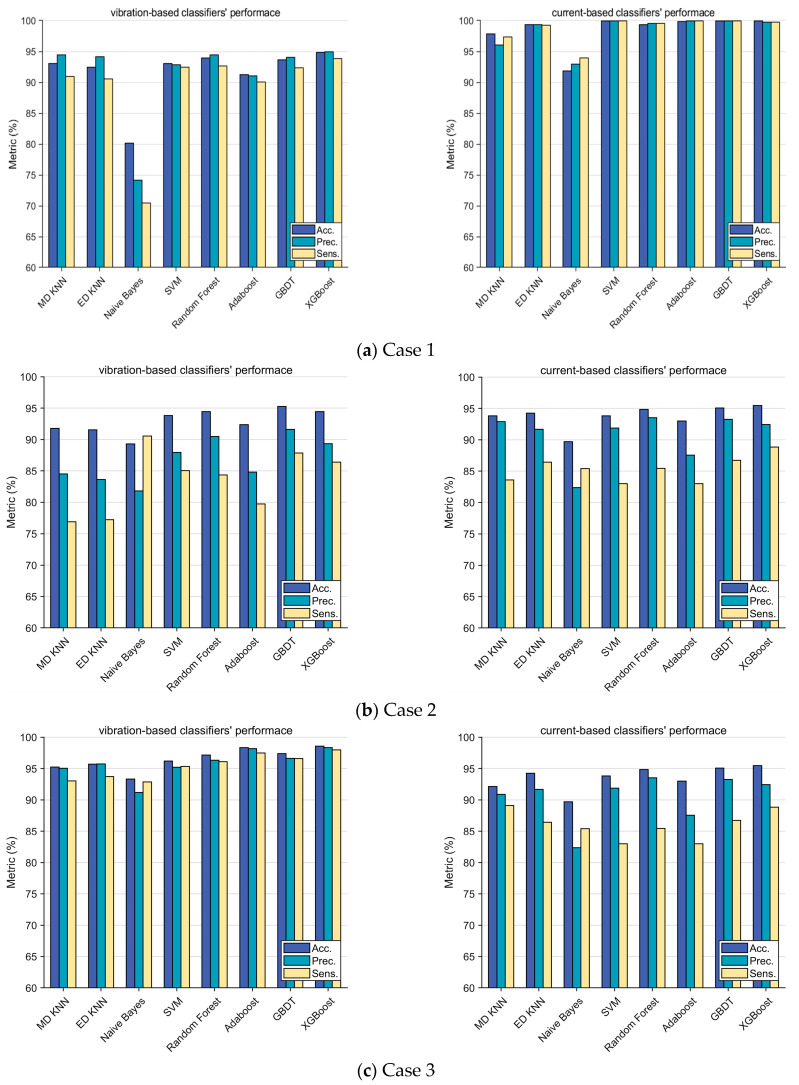

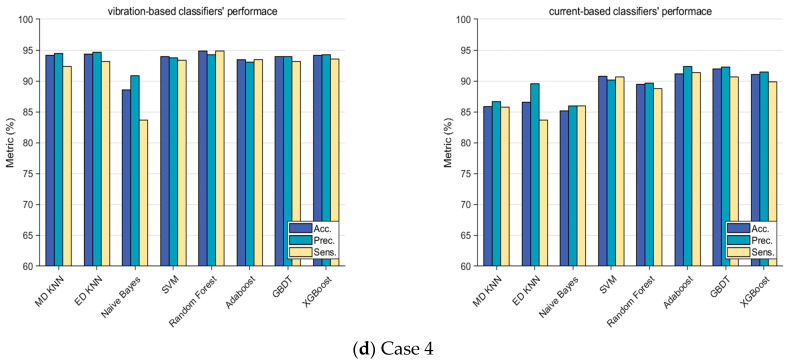
Classifiers’ performance for each case.

Accuracy, precision, and sensitivity are commonly used representative scores to evaluate classifiers. They can be calculated as

(46) $$\mathrm{Accuracy}=\frac{\mathrm{TP}+\mathrm{TN}}{\mathrm{TP}+\mathrm{TN}+\mathrm{FP}+\mathrm{FN}}$$

(47) $$\mathrm{Precision}=\frac{\mathrm{TP}}{\mathrm{TP}+\mathrm{FP}}$$

(48) $$\mathrm{Sensitivity}=\frac{\mathrm{TP}}{\mathrm{TP}+\mathrm{FN}}$$

where *TP* is true positive, *TN* is true negative, *FP* is false positive, and *FN* is false negative. In this study, these scores are used to evaluate the performance of both vibration-based classifiers and stator current-based classifiers for the following four cases, as shown in Figure 20.

Case 1: Operation without cavitation of four combined classes, namely the hump instability range, off-design operation range below BEP, preferred operation range, and off-design operation range above BEP.

Case 2: Cavitation at 0.8*Q_d_* of three combined classes (non-cavitation, cavitation inception, fully developed cavitation).

Case 3: Cavitation at 1.0*Q_d_* of three combined classes (non-cavitation, cavitation inception, fully developed cavitation).

Case 4: Cavitation at 1.2*Q_d_* of three combined classes (non-cavitation, cavitation inception, fully developed cavitation).

For case 1, it can be found that the current-based classifiers demonstrate significantly better performances than the vibration-based classifiers. The performance scores using stator current are mostly above 95%. Therefore, the stator current is more recommended to be used for off-design operation detection. For case 2, the performances of the vibration-based classifiers are relatively similar to those of the current-based classifiers. For case 3, the performance of vibration-based ensemble classifiers, mainly random forest, Adaboost, GBDT, and XGBoost perform better than other vibration-based classifiers and all the current-based classifiers. For case 4, it should be noted that no current-based classifier achieves good results; GBDT is the best (accuracy: 91.13%), which can hardly be called satisfaction. Most of the vibration-based classifiers perform much better. In this sense, information fusion and data compression are considered necessary parts of the proposed diagnosis methodology. In Figure 20, a visual depiction of the newly extracted three-dimensional fusion features space achieved through the use of the t-SNE is presented.

**Figure 21:**
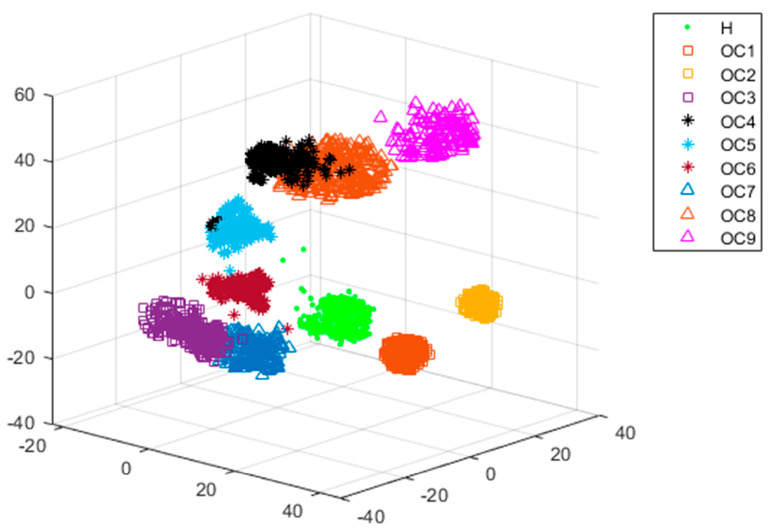
Three−dimensional t-SNE projection achieved in all considered conditions.

In Figure 21, it can be appreciated that different clusters appear to be distinct, and each cluster represents a specific operation condition of the centrifugal pump, namely H (health status: preferred operation range), OC1 (hump instability range), OC2 (operation range below POR), OC3 (operation range above POR), OC4 (cavitation inception at 1.0*Q_des_*), OC5 (critical cavitation at 1.0*Q_des_*), OC6 (cavitation inception at a small flow rate), OC7 (critical cavitation at a small flow rate), OC8 (cavitation inception at a large flow rate), and OC9 (critical cavitation at a large flow rate). The preferred operation range is well separated from others, which means the healthy condition of the centrifugal pump can be assured. In this research, the original hybrid feature space is reduced to five dimensions.

### 6.4. Results Overview and Further Applications

Table 3 shows the classifiers’ performance in all considered conditions. In Table 3, the models based on stator current–vibration fusion outperform the model based on one single source in terms of accuracy, precision, and sensitivity. This means the analyses of vibration and stator current can be complementary, and their combination can provide maximum reliable off-design operation and cavitation detection results for the centrifugal pump.

It should be noted that this research is limited to pumps operating at constant speeds. Future work will be proposed to address variable speed operation.

## 7. Conclusions

This paper discusses the detection of off-design operation and cavitation for centrifugal pumps using accelerometers and current sensors, which is suitable for centrifugal pumps that lack flowmeters and pressure sensors. In this research, a machine learning-based off-design operation and cavitation detection method is proposed. A centrifugal pump is tested considering different operation ranges and different degrees of cavitation. A three-axis accelerometer and three Hall-effect current sensors were used to collect vibration and stator current signals. The vibration signals are processed using the improved wavelet threshold function. A set of time-domain indicators, frequency-domain indicators, and entropy-based indicators are extracted to generate the vibration-based input matrix for machine learning. Meanwhile, the square of the Park vector modulus is used as a function of the stator current signals for power-line demodulation. The RMS indicator, the harmonic indicator, the energy ratios of different IMF components obtained by VMD, and the energy ratio between the feature bands of the marginal spectrum are selected as the current-based indicators. Indicators extracted from both types of signals are proven to be effective for the diagnosis of the centrifugal pump.

Eight classifiers are used to perform machine learning. The majority of classifiers identify the operation conditions of the centrifugal pump correctly. Most ensemble classification algorithms perform better than other single-model classification algorithms. By comparing the classification performance based on different types of signals, it is found that the stator current performs better than the vibration in detecting off-design operations. On the contrary, the vibration performs better in detecting cavitation at the design flow rate and high flow rates. The classifiers based on the fusion of vibration and stator current signals achieved through the use of t-SNE offer higher accuracy, precision, and sensitivity than the classifiers based on one single signal source. In particular, the XGBoost classifier based on the fusion of vibration and the stator current source achieves the highest accuracy of 98.55%. The Adaboost classifier based on the fusion of vibration and the stator current source achieves the highest precision of 98.43%. The SVM classifier based on the fusion of vibration and the stator current source achieves the highest sensitivity of 97.48%. This means the analyses of vibration and stator current signals can be used as a complementary tool for achieving the most reliable detection of off-design operation and cavitation in centrifugal pumps.

## Figures and Tables

**Table 1:** Main parameters of the centrifugal pump and the induction motor.

Device	Parameter	Value
Y160M-2 B3 three-phase asynchronous motor (Manufacturer: Shanghai Qisheng Machinery Equipment Co., Ltd.; Shanghai; China)	Rated voltage	380 V
	Rated speed	2980 rpm
	Rated power	15 kW
	Efficiency	89.4%
	Power factor	0.8
IS-65-50-160-00 centrifugal pump	Impeller inlet diameter	74 mm
	Impeller outlet diameter	174 mm
	Blade width	12 mm
	Blade number	6
	Rated flow	50 m^3^/h
	Rated head	34 m
	Rated speed	2980 rpm
	Efficiency	72.8%
	Specific speed	0.8

**Table 2:** Main parameters of the sensors used in the experiment.

Device	Parameter	Value
SGDN-50 torque transducer	Measurement range	0 ± 50 N∙m
	Frequency output	5–15 kHz
	Precision	0.3%
WBI021F27-1.0 Hall-effect current sensors	Measurement range	AC/DC 0–40 A
	Response time	10 μs
	Precision	1.0%
356A02 accelerometer	Measurement range	±500 g pk
	Sensitivity	10 mV/g
	Frequency range (±5%)	1–5000 Hz
	Broadband resolution	0.0005 g rms
LDG-SIN-CN65-Z2 electromagnetic flowmeter	Measurement range	0–100 m^3^/h
	Precision	0.5%
WIKA S-10 pressure sensors	Measurement range	Inlet: 0–1.6 bar/Outlet: 0–4 bar
	Precision	0.2%

**Table 3:** Classifiers’ performance in all considered conditions.

	Vibration-Based Classifiers	Current-Based Classifiers	Information Fusion-Based Classifiers
(**a**) Accuracy			
MD-KNN	90.95%	91.59%	91.91%
ED-KNN	91.32%	92.35%	92.72%
Naive Bayes	89.51%	90.39%	92.94%
SVM	93.07%	93.24%	98.03%
Random Forest	92.82%	93.61%	97.34%
Adaboost	93.78%	94.57%	98.40%
GBDT	93.07%	93.88%	98.06%
XGBoost	93.70%	94.15%	98.55%
(**b**) Precision			
MD-KNN	92.52%	88.87%	92.92%
ED-KNN	91.98%	92.43%	94.35%
Naive Bayes	89.20%	89.86%	92.18%
SVM	92.90%	92.50%	98.15%
Random Forest	92.30%	93.00%	97.43%
Adaboost	93.98%	94.50%	98.43%
GBDT	92.88%	93.05%	97.63%
XGBoost	93.33%	93.68%	98.35%
(**c**) Sensitivity			
MD-KNN	86.35%	87.60%	88.89%
ED-KNN	86.55%	89.64%	89.32%
Naive Bayes	92.61%	88.22%	95.66%
SVM	91.84%	89.32%	97.48%
Random Forest	91.43%	90.44%	96.31%
Adaboost	92.24%	91.71%	97.23%
GBDT	91.34%	91.71%	97.05%
XGBoost	92.39%	91.66%	97.22%

## Data Availability

Data is unavailable due to privacy.
